# Does sperm quality and DNA integrity differ in cryopreserved semen samples from young, adult, and aged Nellore bulls?

**DOI:** 10.1186/s12610-017-0056-9

**Published:** 2017-06-21

**Authors:** J. T. Carreira, J. T. Trevizan, I. R. Carvalho, B. Kipper, L. H. Rodrigues, C. Silva, S. H. V. Perri, J. R. Drevet, M. B. Koivisto

**Affiliations:** 1 Instituto Federal de Minas Gerais, IFMG, 05, Fazenda Varginha, Estrada Bambuí-Medeiros, Km 05, CEP38900-000 Bambuí, Minas Gerais Brazil; 2 FMVA, Faculty of Veterinary Medicine, UNESP – Univ Estadual Paulista, São José do Rio Preto, Brazil; 3 CRVLagoa, Sertãozinho, Brazil; 4 GReD Laboratory, CNRS UMR6293 – INSERM U1103 – Clermont Université, Clermont-Ferrand, France

**Keywords:** Sperm DNA, Age, Nellore, SCSA, CMA3, 8-OHdG, ADN spermatozoïdaire, Age, Nellore, SCSA, CMA3, 8-OHdG

## Abstract

**Background:**

In humans, it is now well documented that rising paternal age is correlated with decreased sperm DNA integrity and embryonic developmental failures. On the other side of the coin, it is also reported that very young fathers such as teenagers carry an increased risk of adverse birth outcomes. These observations suggest that, at least in humans, there is an age window for optimal sperm DNA integrity. In bovine, little is known about sperm DNA quality in young bulls and how it evolves with age. This study aimed to fill in this gap as it may be of importance for the bovine industry to know when exactly a bull is an optimal performer for reproductive programs.

**Methods:**

Forty Nellore bulls were divided into three age groups: 1.8 to 2 years – young bulls; 3.5 to 7 years – adult bulls; and 8 to 14.3 years – aged bulls. Three ejaculates were collected from each bull, cryopreserved and evaluated for various parameters including: computer-assisted sperm analysis (CASA), plasma membrane and acrosome integrity, mitochondrial potential, sperm nuclear protamination, DNA oxidative damage, and Sperm Chromatin Structure Assay (SCSA).

**Results:**

We report here that young bulls presented superior values for motility, plasma and acrosomal membrane integrity, and high mitochondrial potential. However, they also presented higher values for sperm morphological abnormalities compared to adult and aged animal groups (*p* < 0.05). In addition, young bulls exhibited more defective protamination than older animals did. The oldest bulls showed more nuclear oxidative damage than the younger groups of bulls while both the young and aged groups were found more susceptible to DNA denaturation as revealed with the SCSA test (*p* < 0.05).

**Conclusion:**

These results indicate that young bulls spermatozoa best survived the freezing procedure, followed by adult and aged bulls. However, young and aged bulls were found to be more susceptible to DNA damage, respectively caused by protamine deficiency and oxidation. Therefore, although young bulls have correct semen parameters according to classical evaluation, our results indicate that they may show some structural nuclear immaturity.

## Abstrait

**Contexte:**

Chez l’homme, de nombreuses données indiquent maintenant que l’avancée de l’âge du père est associée à une réduction de l’intégrité de l’ADN des spermatozoïdes et aux échecs de développement embryonnaire. D’un autre côté, il est aussi rapporté que les jeunes pères, tels les adolescents, sont porteurs d’un risque accru d’issue défavorable de la grossesse. Ces observations suggèrent que, au moins chez l’humain, il existe une tranche d’âge dans laquelle l’intégrité de l’ADN des spermatozoïdes est optimale. Chez les bovins, on dispose de peu de connaissances sur la qualité de l’ADN des spermatozoïdes des jeunes taureaux et sur son mode d’évolution avec l’âge. La présente étude a pour but de combler ce manque car il peut être important, pour l’industrie bovine, de savoir à quelle période précise un taureau est un reproducteur optimal pour les programmes de reproduction.

**Matériel et méthodes:**

Quarante taureaux Nellore ont été répartis en trois groupes d’âge: 1, 8 à 2 ans – jeunes taureaux; 3,5 à 7 ans – taureaux adultes; et 8 à 14,3 ans – taureaux âgés. Trois éjaculats ont été collectés par taureau, cryopréservés et évalués pour différents paramètres incluant l’analyse assistée du sperme par ordinateur (CASA), l’intégrité des membranes plasmique et acrosomique, le potentiel mitochondrial, la protamination du noyau, l’altération oxydative de l’ADN et l’évaluation de la structure de la chromatine du noyau du spermatozoïde (SCSA).

**Résultats:**

Nous rapportons ici que les jeunes taureaux présentent des valeurs supérieures de la mobilité des spermatozoïdes et de l’intégrité des membranes plasmique et acrosomique, ainsi qu’un potentiel mitochondrial élevé. Cependant, les jeunes taureaux présentent aussi des valeurs plus élevées d’anomalies morphologiques des spermatozoïdes que celles des groupes adulte et âgé (*p* < 0.05). De plus, les jeunes taureaux ont une protamination plus défectueuse que celle des taureaux plus âgés. Les taureaux les plus âgés présentent plus d’altérations oxydatives du noyau que les jeunes taureaux alors que les deux groupes -jeunes et âgés- sont plus susceptibles d’avoir une dénaturation de l’ADN nucléaire comme indiqué par le SCSA (*p* < 0.05).

**Conclusions:**

Ces résultats indiquent que les spermatozoïdes des jeunes taureaux survivent le mieux au processus de congélation, suivis par les adultes puis les âgés. Toutefois, les jeunes taureaux et les âgés sont plus susceptibles d’avoir une altération de l’ADN, causée respectivement par une protamination déficiente et une oxydation. Par conséquent, bien que les jeunes taureaux aient des paramètres spermatiques corrects à l’évaluation classique, nos résultats indiquent que leurs spermatozoïdes peuvent présenter un certain degré d’immaturité structurale nucléaire.

## Background

DNA damage in human spermatozoa has been associated with a variety of important outcomes including infertility [1], an increased incidence of abortions, and an increased risk of diseases in the offspring [2, 3]. Sperm DNA damage has many faces and it encompasses several distinct situations that may be or not associated in a single sperm cell. Defective protamination, impaired protamine disulphide cross-linking, or/and increased single or double strand breaks resulting from physical/chemical alterations will all lead to abnormal sperm nuclear condensation. Mammalian species are not identical when it comes to sperm nucleus organization and susceptibility to damage. This is rather well illustrated by the observation that mammalian sperm are not equally able to sustain the stress associated with cryopreservation. In this respect, bulls appear to have a rather solid sperm nucleus/chromatin showing a good capacity to sustain the freezing/thawing processes as well as low levels of DNA fragmentation [4–8].

Sperm nuclear organization is very peculiar compared to somatic cells. It results from a long and complex process initiated during the late phases of spermatogenesis, essentially through spermiogenesis and completed when sperm cells go through the epididymis. In humans, it was reported that aging affect these processes resulting in a reduction in sperm chromatin organization which are associated with increased sperm morphological abnormalities and reduced motility. If the decrease in sperm quality associated with age is well documented it is not yet clear whether a certain immaturity of sperm structures and functions is suspected in young mammals. In young rats, Zubkova et al. (2005) reported a defect in protamination and a reduced level of disulphide-bridging on the cysteine-containing protamines when compared to adult animals [4]. Although the animals were found fertile it was questioned whether this poorer state of nuclear maturity could affect offspring health [4].

For large domestic animals, with the help of genomic selection programs, there is a tendency to put into reproduction very young animals. Therefore, it is important to know whether young sexually mature animals present sperm characteristics that are at they optimum in terms of fertilizing capacity. Studies evaluating how aging may affect sperm chromatin integrity in bovine species and the source of the damage are scarce. One study in which semen quality in young bulls and again later in the same mature bulls were evaluated found consistent improvement in semen parameters including sperm DNA integrity, morphology and percentage of intact acrosomes upon aging [9]. In a more recent study, no difference in chromatin integrity as assessed using the sperm chromatin structure assay (SCSA) was found in post-pubertal bulls with mean ages of 13, 18, and 24 months [10]. However, currently, animals with reproductive potential are selected from even younger bulls. Therefore, evaluating seminal characteristics, DNA integrity and mostly the basis of the damage (protamination, oxidative stress) in very young animals versus older animals is still an important issue especially for the bovine artificial insemination industry [11]. This is the goal of the present study in which spermatozoa from young, adult and aged bulls were analyzed and compared.

## Methods

### Animals and semen collection

A total of 40 *Bos indicus* bulls (Nellore) were selected from an artificial insemination center located in the southeast of Brazil (21°04′52″S, 48°02′24″W). The bulls were kept on their native pasture (*Cynodon plectostachyus*) and were given dietary supplementation to optimize their energy balance. Three ejaculates were collected from each bull using an artificial vagina according to a regular twice-a-week collection schedule between September and February of 2010. The samples were cryopreserved (TRIS-egg yolk extender, 7% glycerol in 250 μL French mini-straws) in liquid nitrogen until the evaluation. The 40 selected animals were divided into three groups according to their ages at collection time: 1.8 to 2 years (young group, 27 samples), 3.5 to 7 years (adult group, 57 samples), and 8 to 14.3 years (aged group, 36 samples).

### Flow cytometry

Flow cytometry analysis was performed on an Attune® apparatus (Applied Biosystems by Life Technologies, Grand Island, NY, USA) equipped with 488 nm and 405 nm laser beams and the following emission filters: BL1 530/30 nm “band pass” (BP), BL2575/24 nm BP, BL3 640 nm “long pass” (LP), and VL1 450/40 nm BP, VL2522/30 nm BP, and VL3 603/48 nm BP.

### Computer assisted semen analysis (CASA)

Frozen semen samples (250 μl straws) were thawed at 35 °C for 20 s. Microscopic evaluations were performed using an Olympus BX61 microscope (Olympus, Tokyo, Japan) equipped with phase contrast, differential interference contrast (DIC), and epifluorescence. The Computer Assisted Semen Analysis of sperm movement (CASA 12.3-Hamilton Research®, Beverly, MA, USA) used the IVOS “hardware” (Integrat Visual Optical System, Hamilton Thorne Biosciences®, Beverly, MA, USA). The following kinetic spermatic parameters were evaluated in 2 μl sample aliquots on a slide with four chambers (Leja, NL): MOT (total motility), MP (progressive motility), VAP (average path velocity), VSL (straight-line velocity), and VCL (curvilinear velocity). The fields were randomly selected for each analysis based on the absence of artifacts such as bubbles, particles, or other elements that could adversely affect the evaluation.

### Sperm morphology

Sperm alterations were classified according to Blom (1973) [12] and Barth & Oko (1989) [13] into major defects (i.e., acrosome defects, proximal droplets, abnormal loose heads, abnormal contour, abnormal midpiece, vacuoles, double forms, dag defect) and minor defects (i.e., small normal heads, normal loose heads, abaxial implantation, coiled tails, distal droplets). The abnormalities were additionally grouped as head defects, tail defects (abnormal mid-piece, other tail defects), droplet defects (proximal droplet, distal droplet), and normal loose heads. A total of 200 cells (%, DIC microscopy, oil immersion, 1000×, Olympus BX61) were examined per sample, and one abnormality was recorded per cell.

### Evaluation of membrane integrity - PI and FITC-PSA

One straw *per* batch was thawed (35 °C/20 s). The semen sample was then diluted to 2.10^6^ sperm in 200 μl of PBS (phosphate buffer solution) and stained with 3 μL (14 μM) of propidium iodide (PI) and 50 μL (12.5 μM) of *Pisum sativum* agglutinin conjugated to fluorescein isothiocyanate (FITC-PSA). Samples were incubated at 37 °C for 8 min in the dark and analyzed in an Attune® flow cytometer with a 488 nm argon ion laser excitation beam and simultaneous BL1 (530/30) and BL3 (640LP) readings. The positive control semen samples were subjected to three cycles of “Flash Frozen” [14] prior to staining to promote membrane injury and positive staining in red for PI and green for FITC-PSA.

### Evaluation of mitochondrial potential - JC-1

*One* straw with frozen semen was thawed (35 °C/20s) and diluted in PBS to obtain 2.10^6^ sperm in 200 μl; 6 μL of JC-1 (4 μM) was added to this aliquot, incubated for 8 min at 37 °C in the dark, and analyzed using a flow cytometer with a 488 nm argon ion laser excitation beam and simultaneous BL1 (530/30 nm) and BL2 (575/24 nm) readings. The control cells (with low mitochondrial potential) were incubated with 10 μM of carbonyl cyanide-m-chlorophenylhydrazone (CCCP, Sigma, St. Louis, MO, U.S.A.) at 37 °C for 30 min and stained with JC-1, according to the protocol described previously. CCCP is an uncoupling agent that rescues protons and depolarizes the mitochondrial membrane [15].

### Evaluation of protamination – CMA3 assay

Sperm samples were diluted in 200 μl of McIlvaine’s buffer (2 × 10^6^ sperm) and centrifuged at 300 g for 5 min; the supernatant was discarded, and the sample was resuspended in 200 μl of Carnoy solution (3:1 methanol: acetic acid) and incubated at 4 °C for 5 min. Samples were washed twice with PBS and incubated for 60 min at room temperature with 200 μL chromomycin A3 (CMA3) staining solution (0.4 μM). Samples were washed, resuspended in 1 ml of PBS, and read in the flow cytometer at 488 nm and BL2 filter (575/24 nm); 10,000 events corresponding to sperm were assessed. Semen samples used as positive controls were thawed and centrifuged in McIlvaine’s buffer at 300 g for 5 min, resuspended in PBS containing 5 mM dithiothreitol (DTT) and triton X-100 0,1%, and incubated for 15 min at 37 °C to break disulfide bridges between protamine chains and permeate the cell membrane. Samples were washed twice to remove the detergent solution and DTT, fixed in a Carnoy solution for 45 min [16], stained as described above and read using a 488 nm argon ion laser excitation beam and BL1 filter (530/30 nm); 10,000 events were assessed.

### DNA oxidative – 8-OHdG assay

Guanine oxidation was evaluated using the protocol described by DeIuliis et al. (2009) [17]: thawed semen samples were centrifuged in PBS (300 g), resuspended in 100 μL of DTT (2 mM), and incubated at 37 °C for 45 min. Samples were subsequently washed with PBS, with the addition of 100 μL of 4% paraformaldehyde *per* 100 μL of PBS, and incubated at 4 °C for 15 min. Cells were washed in PBS, and incubated in 100 μL Triton ×100 0,1% at room temperature for 15 min; cells were then prepared according to the OXYDNA® kit (Biotrin, Ireland) procedure, diluted in distilled water at 1:25, and incubated in 1:9 anti-8-OHdG antibody labeled with FITC solution at room temperature for 60 min. Stained samples were washed, resuspended in 1 mL of PBS, and read with a 488 nm argon ion laser excitation beam and BL1 filter (530/30 nm); 10,000 events were assessed. Positive control samples were incubated in DDT (2 mM) for 45 min and were further incubated for 60 min, with the addition of hydrogen peroxide (2 mM) and ferrous sulfate (1 mM).

### Chromatin integrity - SCSA

Sperm samples were adjusted to 2 × 10^6^ spermatozoa/mL in PBS to a final volume of 200 μL, to which 400 μl of acid detergent solution (0.08 M HCl, 15 M NaCl, and 0.1% Triton ×100, pH 1.2) was subsequently added. After 30 s of incubation, 1.2 mL of Acridine Orange (AO) solution was added (6 μg of AO *per* mL of buffer – 0.037 M citric acid, 0.126 M Na2HPO4, 1.1 M EDTA, and 0.15 M NaCl, pH 6.0). The samples were read 3 min after the addition of acid detergent, and 10,000 events were assessed [18]. The assessments were analyzed using the Flowjo® program (Tree Star, Inc., San Carlos, CA, USA) to obtain the total percentage of sperm with damaged chromatin (% DFI). Positive control samples were incubated in DDT (2 mM) for 45 min. Hydrogen peroxide (2 mM) and ferrous sulfate (1 mM) were then added, and samples were incubated for 60 min.

### Statistical analysis

Our null hypothesis (H0) was that there were no differences in DNA integrity and types of chromatin damage in cryopreserved sperm from bulls at different ages. The statistical analyses were performed using the SAS software, “Statistical Analysis System” software (release 9.2. SAS Institute Inc., Cary, NC, USA, 2008). The percentage data were transformed into arcsine to obtain a normal distribution. The results were examined using the “two-way” ANOVA (bulls × group). The averages were compared by the Duncan test and were considered significant when *p* < 0.05. Correlation (r) and determination (r2) coefficients and the regression equation were calculated for selected variables [19].

## Results

### Motility, sperm morphology, membrane integrity and mitochondrial potential

Table 1 summarizes the sperm quality traits for the three groups (young, adult, and aged bulls). The mean total (MOT) and progressive motility (PM) values ranged from 38.0 to 67.8% and 22.1 to 36.9%, respectively, and were significantly higher in young bulls when compared to the two other groups (adult and aged animals). For the other motility parameters (average path velocity [VAP], straight line velocity [VSL] and curvilinear velocity [VCL]) it was the reverse since young animals show consistently lower percentages than both adult and aged animals (see Table 1). In addition (Table 2), young animals presented the highest percentage of sperm with high mitochondrial potential (81.7%), and membrane and acrosome integrity (MIAI – 54.7%) compared to the adult (MP 67.82%; MIAI – 46.7%) and the aged groups (MP 65.3%; MIAI-37.5%) (*p* < 0.05). When membrane integrity (via propidium iodide [PI] staining) and acrosome integrity (via PSA-FITC staining) were considered together, the aged group presented the lowest percentage of undamaged cells, while the young animals had the highest percentage. The adult group presented intermediate values (Table 2). Finally, in terms of total morphological sperm defects it is interesting to note that the young bulls group presented the highest percentage of abnormal spermatozoa when compared with the adult and aged animals groups (*p* < 0.05). As shown in Table 3, total motility (MOT), progressive motility (PM), high and medium mitochondrial potential and membrane and acrosome integrity showed a negative correlation with age while damaged membrane and damaged acrosome showed a positive correlation with age.

**Table 1:** Groups of young, adult and aged Nellore bulls with number of evaluated samples (27, 59 and 36, respectively), scrotal circumference and computer assisted sperm analysis after cryopreservation (means ± SD)

Group (n)	Age (years)	Scrotal circumference	MOT (%)	PM (%)	VAP (μm/s)	VSL (μm/s)	VCL (μm/s)
Young (27)	1.9 ± 0.1	38.17 ± 3.50 ^a^	67.80 ± 17.68^a^	36.93 ± 11.28^a^	74.85 ± 12.53^b^	53.41 ± 10.23^b^	144.82 ± 25.48^b^
Adult (57)	4.8 ± 1.0	41.14 ± 2.19 ^b^	39.83 ± 13.52^b^	23.13 ± 8.65	85.80 ± 12.23^a^	68.13 ± 10.89^a^	160.44 ± 23.10^a^
Aged (36)	10.1 ± 2.0	40.13 ± 2.89 ^b^	37.99 ± 17.75^b^	22.12 ± 11.64^b^	82.93 ± 13.29^a^	65.59 ± 12.84^a^	155.66 ± 24.37^ab^

Means with different superscripts same column differ significantly (*p* < 0.05) by the Duncan test

MOT (total motility)

PM (progressive motility)

VAP (average path velocity)

VSL (straight-line velocity)

VCL (curvilinear velocity)

**Table 2:** Sperm quality traits in young, adult, and aged Nellore bulls evaluated after cryopreservation (means ± SD)

Group	High mitochondrial potential	Intact membrane/Acrosome	Damaged membrane	Damaged acrosome	Morphologic defects
Young	81.70 ± 9.40ª	54.70 ± 9.49ª	34.70 ± 12.27^b^	32.52 ± 8.75^c^	18.78 ± 7.02^a^
Adult	67.82 ± 10.38^b^	46.68 ± 10.13^b^	38.81 ± 9.76^ab^	38.23 ± 11.52^b^	14.62 ± 7.80^b^
Aged	65.31 ± 12.12^b^	37.53 ± 9.84^c^	41.76 ± 10.67^a^	45.85 ± 13.72^a^	15.14 ± 6.37^ab^

Means with different superscripts same column differ significantly (*p* < 0.05) by the Duncan test

**Table 3:** Correlation coefficient and *P* values between bovine age groups and total sperm motility (MOT), progressive motility (MP), mitochondrial potential, and membrane and acrosome integrity

Variable	Correlation coefficient	P
MOT	−0.45	<0.0001
PM	−0.37	<0.0001
High mitochondrial potential	−0.35	0.0001
Intact membrane and acrosome	−0.36	0.0001
Damaged membrane	0.25	0.0091
Damaged acrosome	0.19	0.042

### Protamination, oxidative damage, chromatin integrity

Table 4 shows the results of the sperm nuclear and chromatin integrity investigations. Regarding sperm protamination assessment, the group of young bulls showed a greater percentage of CMA 3-positive sperm cells than did the adult and aged groups (*p* < 0.05). Since CMA-3 staining is inversely correlated with the level of sperm nuclear condensation, this observation suggests that the sperm nucleus of young bulls is significantly less condensed than the sperm nucleus of adult and aged animals. When investigating DNA fragmentation indirectly using the acridine orange SCSA staining assay we found that both the young and the aged groups show the highest DNA fragmentation index (DFI) while the adult group was significantly lower (Table 4, DFI column). Concerning sperm DNA oxidative damage as evidenced by using an anti-8-OHdG detection system (OXIDNA assay) we found that this parameter increased with the age groups reaching its maximum value in the aged animals (Table 4, column 8-OHdG). A correlation analyses brought forward that CMA3 data were negatively correlated with age while 8-OHdG data were positively correlated with age. No statistical valid correlation with age was found for the SCSA data.

**Table 4:** Correlation between age and DNA integrity; mean and standard deviation of percentages of sperm cells with chromatin alterations detected by the CMA3, 8-OhdG and SCSA techniques of young (*n* = 27), adult (*n* = 59), and aged (*n* = 36) bovine semen samples

Group	CMA3	8-OHdG	SCSA (%DFI)
Young	1.57 ± 0.76ª	25.69 ± 6.29^c^	3.55 ± 0.61ª
Adult	1.09 ± 0.63^b^	34.89 ± 9.20^b^	2.13 ± 0.78^c^
Aged	0.90 ± 0.59^b^	44.11 ± 11.00^a^	3.09 ± 0.96^b^
Correlation (*P* value)	−0.33 (>0.0001)	0.63 (>0.0001)	−0.14 (0.1401)

Means with different superscripts in the same column differ significantly (*p* < 0.05) by the Duncan test

## Discussion

Our observations suggest that young bull sperm samples are characterized by their high motility and that upon aging motility parameters decrease. This is not new, since there are similar reports showing that *Bos Taurus* bulls sperm motility decrease upon aging [20]. It was proposed to be correlated with a decrease in antioxidant protection in the semen upon aging and, consequently, in an increase in oxidative damage to sperm cells that are known to alter sperm movement [21]. Similarly, we did observe that sperm samples from old bulls suffer more oxidative damage than sperm samples from younger animal groups. In good agreement with our observation that spermatozoa from the youngest animals had the best total motility we found that they also exhibited the highest mitochondrial potential. This is somehow logical since mitochondria activity sustains flagellar movement [22, 23]. It must be noted that there are however contradictory reports showing that age does not influence significantly bull sperm motility although they concerned a distinct bull breed (*Bos Indicus*) [24–26]. Looking in more details at our motility data we found that even-though young bulls exhibited the highest total motility (MOT) and progressive motility (PM), they also presented the lowest progressive linear velocity (VSL) as well as the lowest average path velocity (VAP) and curvilinear velocity (VCL), parameters indicating of head lateral movement and hyperactive movement [27, 28] when compared to the other age groups. This may reflect a certain immaturity of the young sperm samples that cannot achieve a complete array of flagellar movements. Therefore, the adult and aged animal groups we investigated showed a better quality of sperm movement even-though these sperm samples appear globally less motile.

Our combined evaluation of sperm plasma membrane/acrosomal membrane integrity showed a regular decrease upon aging. Since we dealt with cryopreserved samples this may be a reflection of their weaker ability to sustain the freezing and thawing protocol that are known to affect internal and external cell membranes essentially through the vicious circle of membranous lipoperoxidative processes [29]. The age-related decrease in sperm and seminal plasma antioxidant protective activities is well documented as well as the impact cryopreservation has on these activities causing a major reduction in glutathione peroxidase and superoxide dismutase [4, 21, 30].

Taken together, and, without surprise, progressive motility, functional mitochondria as well as plasma/acrosomal membrane integrity were found negatively correlated with age. Finally, when it came to sperm morphological alterations our study revealed that the highest percentages of structurally abnormal sperm were found for the young and the aged animal groups. This observation somewhat supports the idea that young bulls may be in a certain state of immaturity evidenced by this higher level of teratospermia.

To analyze more deeply bull sperm characteristics in these three age groups we looked at the sperm nuclear compartment by evaluating sperm DNA compaction, sperm nuclear protamination and sperm DNA oxidation. The rationale behind these investigations was that if abnormal head morphology was a characteristic of young bull spermatozoa this might be due to nuclear alterations as it is supported by several studies [31–34]. To our knowledge there is no other study that has focused on the bull sperm nuclear integrity upon aging. This is of particular importance as the integrity of the sperm nucleus is critical for the success of fertilization, the completion of the developmental program and the quality of life of the progeny. These points are rather important in the bovine industry for the efficiency and the cost effectiveness of insemination programs. Regarding bovine sperm nuclear condensation, Kipper et al. (2017) [34] noted that spermatozoa from young bulls were morphometrically larger compared to those from older bulls and that alterations in chromatin condensation were related to heads with greater diameters. In another study concerning a different model of agricultural interest, Martí et al. (2011) [35] morphometrically evaluated the heads of sperm cells from young goats shortly after reaching sexual maturity and observed heads with larger sizes compared to those of the adult animals, indicating a possible transition period between puberty and full sperm head size maturity. Our present data corroborate these findings since we found a negative correlation between bull age and the percentage of sperm with impaired/diminished protamination. Clearly, spermatozoa from the youngest animal group showed a higher percentage of sperm with defective protamination (ie: with higher CMA3 staining). Defective protamination and/or histone retention in the sperm nucleus explain abnormal nuclear condensation [36]. This lower state of nuclear condensation of young bull sperm was associated with a higher DNA fragmentation index which is a rather logical situation since a less condensed nucleus is more incline to suffer DNA strand breaks. Consisting with our observation, Fortes et al. (2012) [10] also found that younger bulls have higher DNA fragmentation indices (DFI%) compared to adult animals. Similarly to our observation, Zubkova et al. (2005) [4] evaluated protamination in young and old rats sperm and found that young animals showed the highest percentage of sperm with CMA3-positive staining compared to old animals. It is interesting to note that when compared to CMA3 staining levels reported in human sperm, bull CMA3 staining is markedly low [16, 37, 38]. Although, to our knowledge, there is no other study that corroborate our CMA3 readings in bull sperm, this observation may suggest that compared to human sperm the protamination level of bull sperm is rather high. This may explain why bull sperm is so resistant and survive rather well the freezing/thawing processes accompanying cryopreservation and Intra-Uterine-Insemination bovine programs when compared to sperm cells from other species. Regarding oxidative DNA damage, one of the main source of post-testicular damage to spermatozoa [39], we noticed that spermatozoa from the youngest group showed less DNA oxidative alterations as revealed by their reactivity towards an antibody directed toward 8-OHdG residues. On the contrary, older bulls were more reactive and showed more DNA oxidative damage. This finding may appear contradictory since if younger bulls show less condensed sperm nucleus, it might be expected that they should be more sensitive to DNA oxidative damage. This rationale does not take into account the efficiency of the numerous antioxidant actors that are present in the post-testicular compartments (epididymal fluid & seminal fluid) to protect the sperm cells. It is well documented that these systems become less efficient upon aging in all mammals tested so far [40, 41]. This may explain why DNA oxidative damage is not preponderant in spermatozoa of young animals even-though the sperm nucleus is apparently in some state of immaturity.

## Conclusions

In conclusion, our study brings forward that young bulls as well as aged bulls present spermatozoa that are not at their optimal condition in terms of nuclear integrity. In young bulls, deficient protamination and higher DNA fragmentation reveal a state of nuclear fragility or immaturity. In elderly bulls, higher susceptibility to oxidative alterations may explain their intermediate susceptibility to acidic treatment (DFI%) revealing also a certain state of fragility of the sperm nucleus. Although this study does not bring evidence as to the fertility potential of these 3 groups of bulls, which would be an interesting parameter to have, it has the advantage to show that both very young bulls sperm may not be at their optimal in term of nuclear integrity. This is particularly important to take into account because the paternal chromosomal lot is the essence of what must be conveyed by the spermatozoa in the oocyte. An intact paternal genome together with an intact maternal counterpart will drive the developmental program and will determine the quality of life of the offspring. In an industrial perspective, to know that young bulls, although sexually mature by some aspects, do show sperm cells that may be not totally mature is rather an important parameter. It may help to explain the lower reproductive efficiency of some bulls that are used as reproducers too early. It may also avoid misleading conclusions regarding the fertilizing ability of some bulls that may have been tested too early. In fine, based on our observations we propose that it might be of interest to introduce assays aimed at evaluating the integrity of the bull sperm nucleus together with the classical sperm evaluation check-list which includes sperm count, motility and morphological assessments. The results from this study suggest that the early introduction of young animals into artificial insemination centers should be cautiously evaluated because, despite providing satisfactory semen samples according to the traditional evaluation parameters, young animals may harbor a certain degree of nuclear/chromatin immaturity.
